# Rare Variant Association Analysis Reveals *B3GNT9, RANBP10, and C1QL4* As Potential Genes for Cognitive Preservation in the Mid‐Western Amish

**DOI:** 10.1002/alz70855_102970

**Published:** 2025-12-23

**Authors:** Yining Liu, Yeunjoo E. Song, Weihuan Wang, Audrey Lynn, Kristy L. Miskimen, Sarada L. Fuzzell, Sherri D. Hochstetler, Renee A. Laux, Laura J. Caywood, Jason E. Clouse, Sharlene D. Herington, Ping Wang, Alex V. Gulyayev, Leighanne R. Main, Daniel A. Dorfsman, Noel C. Moore, Michael B. Prough, Dawn Miller, Andrew F Zaman, Larry D Adams, Patrice G Whitehead, Paula K. Ogrocki, Alan J. Lerner, Jeffery M Vance, Michael L Cuccaro, William K. Scott, Margaret Pericak‐Vance, Jonathan L Haines

**Affiliations:** ^1^ Department of Population and Quantitative Health Sciences, Case Western Reserve University School of Medicine, Cleveland, OH, USA; ^2^ Department of Population and Quantitative Health Sciences, Institute for Computational Biology, Case Western Reserve University, Cleveland, OH, USA; ^3^ Cleveland Institute for Computational Biology, Case Western Reserve University, Cleveland, OH, USA; ^4^ John P. Hussman Institute for Human Genomics, University of Miami Miller School of Medicine, Miami, FL, USA; ^5^ Department of Genetics and Genome Sciences, Case Western Reserve University School of Medicine, Cleveland, OH, USA; ^6^ University of Miami Miller School of Medicine, Miami, FL, USA; ^7^ Department of Neurology, Case Western Reserve University School of Medicine, Cleveland, OH, USA; ^8^ Department of Neurology, University Hospitals Cleveland Medical Center, Cleveland, OH, USA; ^9^ Department of Neurology, University of Miami Miller School of Medicine, Miami, FL, USA; ^10^ Dr. John T. Macdonald Foundation Department of Human Genetics, University of Miami Miller School of Medicine, Miami, FL, USA; ^11^ Cleveland Institute for Computational Biology, Department of Population & Quantitative Health Sciences, School of Medicine, Case Western Reserve University, Cleveland, OH, USA

## Abstract

**Background:**

Cognitive preservation is observed in older individuals who are at high risk of cognitive decline but remain cognitively unimpaired (CU). Identifying rare variants (RVs) associated with cognitive preservation may address the missing heritability of Alzheimer Disease (AD). Furthermore, RVs are likely to be enriched to higher frequencies through genetic drift in founder populations, making them easier to detect. This study utilized whole genome‐sequencing (WGS) data from the Mid‐Western U.S. Amish to search for RVs and their associated genes that are associated with cognitive preservation.

**Method:**

Our study included 868 Amish individuals (age 62.5‐101.8, mean = 82.6). Of these, 518 were CU and considered as cognitively preserved (mean age 81.5), and 350 were cognitively impaired (CI) (including mild cognitively impaired (mean age 83.6), cognitively impaired but not AD (mean age 85.0), and AD (mean age 84.9)). Cognitive status was determined based on adjudication of neurocognitive and physical exams by clinical experts. Gene‐based genome‐wide RV association tests were conducted against the binary cognitive status (CU vs CI) to test for RVs associated with cognitive preservation. Variants with minor allele frequency (MAF) < 1% in the general European population and MAF < 5% in the Amish were included in the study. To detect RVs in the coding regions, RVs annotated as having high or moderate impact on protein function by SNPEff were aggregated and analyzed using SKAT‐O tests. RVs in the non‐coding regulatory regions were grouped and tested following the STAARpipeline. All association tests accounted for age, sex, study center, and relatedness.

**Result:**

We observed suggestive associations (significant p threshold = 3.96x10^‐6^) of three coding RVs with cognitive preservation mapped in two genes: *B3GNT9* (SKAT‐O *p* = 5.07x10^‐5^) and *RANBP10* (SKAT‐O *p* = 6.59x10^‐5^). All three RVs are missense variants with moderate impact on protein function. In addition, grouping of 14 RVs in the promoter DNAase I hypersensitive site of *C1QL4* revealed another suggestive signal (STAAR‐O *p* = 7.00x10^‐6^, significant p threshold = 4.05x10^‐7^).

**Conclusion:**

Our gene‐based genome‐wide RV association analyses identified suggestive associations with cognitive preservation in the Amish. These results underscore the potential significance of RVs in elucidating the full genetic architecture of AD.

